# Laminin-modified gellan gum hydrogels loaded with the nerve growth factor to enhance the proliferation and differentiation of neuronal stem cells

**DOI:** 10.1039/d0ra01723j

**Published:** 2020-05-01

**Authors:** Wenqiang Li, Anfei Huang, Yanheng Zhong, Lin Huang, Jing Yang, Changren Zhou, Lin Zhou, Yanling Zhang, Guo Fu

**Affiliations:** The First Affiliated Hospital, Jinan University Guangzhou China hyzhoulin@126.com dr.fuguo@qq.com; Engineering Technology Research Center for Sports Assistive Devices of Guangdong, Guangzhou Sport University Guangzhou China; Department of Materials Science and Engineering, Jinan University Guangzhou China; Department of Ultrasound, Third Affiliated Hospital, Sun Yat-sen University The People's Republic of China 15136102@qq.com

## Abstract

The reconstruction of peripheral nerves has lately received great attention as many patients suffer from peripheral nerve injury every year around the world. However, the damage to human nerve cells has different degrees of irreversibility due to a slow growth speed and low adhesion with the surrounding tissues. In an effort to overcome this challenge, we applied novel laminin (LN)-modified thiolated gellan gum (TGG) and loaded the nerve growth factor (NGF) as a tissue engineering scaffold for facilitating neuronal stem cell proliferation *via* a synergy effect for the ERK–MAPK pathway. TGG was characterized by ^1^H NMR spectroscopy and scanning electron microscopy, and its rheological behavior was also studied. The NGF release curve fitted the Korsmeyer–Peppas model and belonged to a Fickian diffusion-controlled release mechanism. The neuronal stem cells from newborn SD rats could adhere tightly and proliferate at a relatively rapid speed, showing excellent biocompatibility and the ability to promote growth in the modified TGG. LN and NGF could decrease the apoptosis effects of neuronal stem cells, as shown *via* the flow cytometry results. In a three-dimensional culture environment, LN and NGF could facilitate neuronal stem cells to differentiate into neurons, as proved by immunofluorescence, q-PCR, and western blot analyses. Therefore, the rational design of the TGG gel loaded with NGF has promising applications in the reconstruction of peripheral nerves.

## Abstract 2

Laminin-modified thiolated gellan gum and loaded with the nerve growth factor in facilitateding neuronal stem cell proliferation and differentiation.

## Graphical abstract

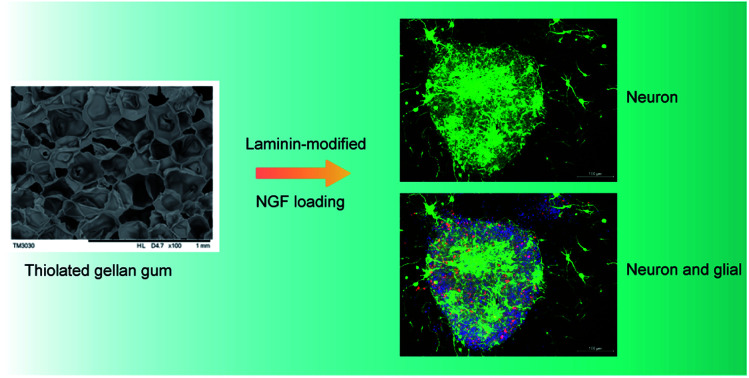

## 1 Introduction

Peripheral nerve injury is a common type of injury in surgery. A large number of patients around the world suffer from peripheral nerve injuries every year, most of which are caused by cutting, burns, or degenerative diseases.^1^ However, the damage to human nerve cells has different degrees of irreversibility due to a slow growth speed and adhesion difficulty with the surrounding tissues. How to cure peripheral nerve injury has become a hot topic in clinical medicine.^2^ In order to solve this problem, numerous medical and scientific workers have performed considerable exploration and research on the repair of peripheral nerve injury; they have made some progress in the oral application or injection of neurotrophic drugs^3^ and nerve growth factor (NGF), gene therapy,^4^ nanotherapy,^5^*etc.* However, this still cannot change the fact that human peripheral nerve regeneration is difficult and the growth is slow, and the repair of peripheral nerve injury still needs our continuous efforts for further development and improvement.^6^ Among the numerous treatments, the use of tissue engineering scaffolds has been spotlighted for supporting the controlled release of neurotrophic factors and enhancing the growth of neurons or neuronal stem cells. Current studies have shown that scaffolds loaded with neurotrophic factors can promote cell adhesion and directional growth.^7,8^ Nerve growth factor (NGF) is one of the earliest and most studied neurotrophic factors. NGF is a glycoprotein, which is mainly distributed in glial cells, Schwann cells, skeletal muscles, *etc.* NGF has many biological functions; its most important and the most relevant function is to protect and promote nerve growth and development.^9,10^ NGF plays an important role in the repair of peripheral nerve injury. Due to the important role of the nerve growth factor in nerve growth, development, and repair, it has received extensive attention and research, and its role in promoting peripheral nerve regeneration and repair has been repeatedly studied and verified. However, the bioactivity of NGF is limited by the blood brain barrier and its short half-life. In addition, a good drug delivery system needs the drug to exist for a long time *in vivo* with low systemic side effects and a high curative effect. Therefore, it is urgent to develop a method to improve NGF utilization.^11^ Tissue engineering applied in curing peripheral nerve injury has become a promising approach in recent years.^12–14^

In this work, we used modified gellan gum (TGG) loaded with laminin (LN) and nerve growth factor (NGF) to enhance the proliferation and differentiation of neuronal stem cells *via* a synergistic reinforcement mechanism. Gellan gum is a kind of anionic linear exopolysaccharide produced *via* aerobic fermentation by *Sphingomonas*.^15^ A gellan gel solution has high transparency, temperature lag, and adjustable melting point. It is used as a food additive and stabilizer because of its excellent gel properties. In recent years, its research and application in the field of medicine have gradually become a topic of interest. Gellan gel has also been applied and researched in the field of medicine, especially in pharmacy and tissue engineering.^16^

The chemical structure of biomaterials is generally an important factor affecting the growth behavior of cell adhesion.^17–19^ LN is one of the most important components of the extracellular matrix of peripheral nerve cells. In the peripheral nervous system, LN is mainly expressed by Schwann cells and is widely distributed in the cell surface; it is an ubiquitous component in the basement membrane.^20^ LN is responsible for the neurite outgrowth promotion activity of the conditioned medium factor.^21^ LN is an important component for peripheral nerve regeneration. Therefore, LN is often combined with other materials (collagen, chitosan, *etc.*) to promote nerve repair in nerve injury.^22,23^

In this work, the ^1^H NMR spectra, SEM image, rheological behavior, and degradation properties of the designed TGG hydrogel were studied to validate the physical properties of the LN-modified TGG. The NGF release curve and kinetics were also measured. For the biological performance, we investigated the proliferation behavior and apoptosis process induced by LN-modified gellan gum hydrogels and explored the synergy mechanism for the ERK–MAPK pathway. In addition, we studied the differentiation behavior *via* immunofluorescence, q-PCR, and western blot analyses. Overall, the prepared TGG gel has promising applications in NGF delivery and can provide new opportunities for peripheral nerve injury treatment in clinical applications.

## 2 Experimental section

### 2.1 Preparation and characterization of TGG

First, thiolated gellan gum (TGG) was produced by a previously reported method.^24^ Then, 1.5% (w/v) TGG was dissolved in deionized water at 60 °C, and the obtained pre-gel solution was injected into a mold to form a gel *in situ* under room temperature conditions.

#### Characterization

TGG was characterized by ^1^H NMR spectroscopy to exactly determine its chemical structure, with the measurement performed on a Bruker 500 MHz Ascend system. The morphology of the hydrogels at 3 wt% concentration was observed by scanning electron microscopy (SEM, LEO1530 VP, Philips, Netherlands). Briefly, the freeze-dried gel specimens were cryogenically fractured under liquid nitrogen and sputter-coated with gold for observation. The rheological measurements of the hydrogels were performed using a rotational rheometer (DHR, TA Instruments, USA) with a parallel plate setup (diameter of 20 mm and a gap of 1 mm). A strain amplitude sweep test (*γ* = 0.001–1000%, *ω* = 6.28 rad s^−1^) and dynamic oscillatory frequency sweep measurements (*ω* = 0.1–100 rad s^−1^, *γ* = 1%) at 37 °C were performed to study the viscoelastic properties of the TGG gels.

#### Experimental group preparation

The biological experiments were performed on four groups: control group (TGG 3 wt%); LN group (TGG 3 wt%, LN peptide density of 1 μM); GG group (TGG 3 wt%, NGF density of 200 ng mL^−1^); LN + NGF group (TGG 3 wt%, LN peptide density of 1 μM, NGF density of 200 ng mL^−1^).

### 2.2 NGF release study

Pre-gel solution was mixed with a concentrated cell suspension at 37 °C. The final content of each component was as follows: TGG 3 wt%, LN peptide density of 1 μM, NGF density of 200 ng mL^−1^ (purchased from Univ-bio Co. Ltd China). Subsequently, the hydrogel was immersed in a PBS solution (pH 7.4) at 37 °C. At the designed time interval, the solution was collected and replaced with fresh PBS. The collected PBS solutions were stored at −20 °C for later analysis. The amount of NGF in the collected PBS solution was measured using Sandwich ELISA following the manufacturer's protocol. First, flat-bottomed 96 well polystyrene plates (Nalge Nunc, Roskilde, Denmark) were coated with 50 μL NGF capture antibody solution and the wells were blocked with a blocking solution containing solutions of 1% BSA (w/v), 5% sucrose, and 0.05% NaN_3_ in PBS (50 μL per well) for 2 h at 37 °C. Second, 50 μL aliquots of NGF test solutions were added to each well after washing the wells twice with TBST (PBS with 0.05% Tween 20) and were then incubated at room temperature for 2 h. Third, 50 μL of anti-NGF-HRP was added to each well and the sample was held for 2 h at room temperature. Finally, TMB (3,3′,5,5′-tetramethylbenzidine) was added as a substrate and the samples were re-incubated for 30 min; the optical density was measured at 450 nm using an ELISA reader. The NGF release kinetic model equation of Korsmeyer–Peppas is as follows:
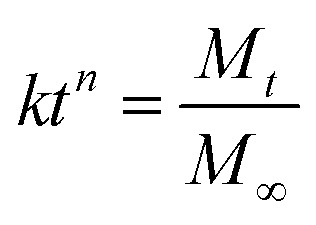


### 2.3 TGG degradation behavior

TGG hydrogel (3 wt%) and TGG/LN/NGF hydrogel (TGG 3 wt%, LN peptide density of 1 μM, NGF density of 200 ng mL^−1^) were prepared as described above in Section 2.1. Then, the hydrogels were freeze-dried for weighing. The same quantity of TGG and TGG/LN/NGF dried hydrogels was put into PBS solution and then placed in a shaker (50 rpm, 37 °C). Every three days, the hydrogels were taken out and freeze-dried to weigh the quality. Finally, all the group and weight data were considered together to calculate the weight loss ratio.

### 2.4 Neuronal stem cell culture

SD rats 14 days pregnant were weighed and given abdominal anesthesia with 10% chloral hydrate in proportion. After anesthesia, they were soaked in 75% ethanol for 5 min. After the rats were fixed, the operation was carried out on a laminar flow operating table. The cerebral cortex of fetal rats was removed under aseptic conditions and put into a pre-cooled aseptic DMEM/F12 culture medium. The removed cerebral cortex was cut into 1 mm × 1 mm × 1 mm size using scissors. After the single-cell suspension was collected, it was centrifuged at 800 rpm for 10 min, the supernatant was discarded, and the prepared neural stem cell culture medium was added to resuspend the cells. The cell density was adjusted to 1 × 10^5^ mL, and the cells were inoculated into 50 mL culture bottles, each of which was inoculated to about 10 mL, and cultured in a humidity incubator at 37 °C with a volume fraction of 5% CO_2_.

Pre-gel solution was mixed with a concentrated cell suspension at 37 °C. The experiment was divided into four groups: TGG control group, LN group, NGF group, LN + NGF group (the detailed concentrations are consistent with those detailed in Section 2.1). Subsequently, the cell-loaded hydrogel was pipetted into the cell culture plate and cultured under the same conditions. Cell viability was manually counted *via* CCK-8 detection and the proliferation morphology was observed by fluorescence microscopy at 72 h (Lumascope 460, Etaluma).

For the differentiation study, after 1 week culture, each specimen was fixed in 4% paraformaldehyde, permeabilized by 0.1% Triton X-100, blocked with bovine serum albumin, and immune-stained with βIII–tubulin (1 : 100) for neuron cells and GFAP (1 : 500) for glial cells and 4′, 6-diamidino-2-phenylindole (DAPI, Life) for the nucleus. Then, confocal laser scanning microscopy (CLSM, CarlZeiss LSM880 META, Germany) was performed.

### 2.5 Western blot assay

The cells were cultured with the TGG control group, LN group, NGF group, and LN + NGF group, harvested and then boiled in sample loading buffer for 10 min (95 °C). The proteins were electrophoretically resolved on a 12% SDS-PAGE gel at 120 V. The proteins were electrophoretically transferred to PVDF membranes at 350 V for 90 min (Millipore, USA) and incubated with 5% fat-free milk-TBST buffer. Then, the PVDF membrane was blotted with primary antibodies overnight at 4 °C in TBST buffer. The membrane was washed with TBST three times for 10 min each time. The membranes were then incubated with peroxidase-conjugated secondary antibodies and subsequently washed with TBST three times for 10 min each time. The chemiluminescence signal was developed to visualize the proteins according to the manufacturer's instructions. Primary antibodies targeting the following proteins were used: p-ERK1/2 (ab214362), ERK1/2 (ab17942), p-MEK1 (ab96379), MEK (ab32576), βIII-tubulin (ab18207), GFAP (ab7260).

### 2.6 Cell apoptosis assay

Neuronal stem cells were seeded in six-well plates (5 × 10^5^ cells per well) with the plate substrate at the bottom of the GG control group, LN group, NGF group, and LN + NGF group. Neuronal stem cells were then trypsinized, centrifuged, washed with PBS, and stained with 5 μL Annexin V (1 vol%) and 5 μL propidium iodide (PI) (1 vol%) following the operating instructions. Neuronal stem cells were quantified using flow cytometry (FACS Gallios, Beckman, USA).

### 2.7 Statistical analysis

All of the quantitative data were expressed as the mean ± standard deviation (SD). Two treatment groups were compared by Student's *t*-test. GraphPad Prism version 8.0 (GraphPad Inc., La Jolla, CA, USA) was used for statistical analyses. The results were considered statistically significant when *p < 0.05 and **p < 0.01. The release curve model was simulated by Origin 2017.

## 3 Results and discussion

### 3.1 Characterization of the hydrogel materials

**Fig. 1:**
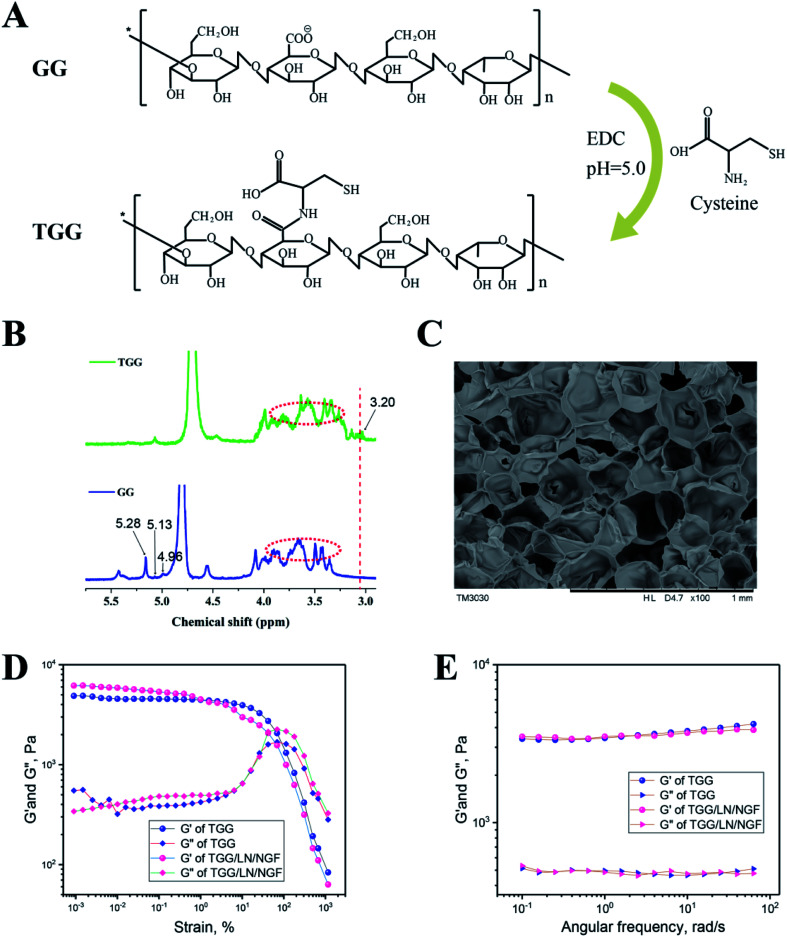
(A) The synthetic procedure for TGG. (B) ^1^H NMR spectra of GG and TGG. (C) SEM image of TGG hydrogel at the concentration of 3 wt%. The storage modulus (G′) and loss modulus (G′′) were plotted logarithmically against the strain (D) and frequency (E) of TGG and TGG/LN/NGF at 37 °C.

GG was modified to TGG for improving the biological properties, and the synthetic procedure for fabricating TGG, shown in Fig. 1A, is a previously reported method.^24^ The ^1^H NMR spectra of TGG and GG are shown in Fig. 1B. The spectrum of GG indicates the presence of three characteristic peaks corresponding to –CH of rhamnose (*δ* 5.28 ppm), –CH of glucuronic acid (*δ* 5.13 ppm), and –CH of glucose (*δ* 4.96 ppm) together with a signal for carbohydrate nonterminal protons at 3.915–3.363.^25^ For the TGG characteristic peaks, it can be seen that a weak peak at 3.20 ppm represents the protons from the –CH_2_– groups connected to the α-carbon in cysteine,^26^ revealing that the thiol group was successfully integrated into GG. Fig. 1C shows the SEM image of the cryo-fracture morphology of the TGG hydrogel, exhibiting an open porous network structure and core interconnectivity beneficial to the transfer of nutrients for cells, due to which it can be regarded as a drug-delivery vehicle.^27^ To investigate the viscoelastic behaviors of the TGG and TGG/LN/NGF hydrogels, rheological tests were carried out. The results of strain amplitude sweep tests are shown in Fig. 1D, which displays a broad linear viscoelastic region and great anti-shear ability; there was merely a network collapse at high strains over 80%, indicating a wide processing range and good shear thinning (injectable). Frequency-dependent oscillatory shear rheology (Fig. 1E) of the corresponding hydrogel sample showed that the G′ and G′′ values of the tested samples did not cross with the increase in the angular frequency, demonstrating the formation of a stable network structure of the hydrogel. The consistent mechanical and rheological curves of the TGG and TGG/LN/NGF hydrogels revealed that the addition of LN and NGF did not damage the mechanical properties of the hydrogel.

### 3.2 NGF release behavior and hydrogel degradation

**Fig. 2:**
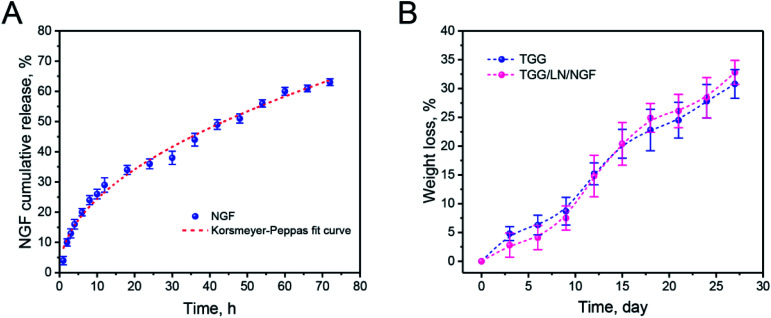
The release curve of NGF from TGG (blue solid point) and Ritger–Peppas-fitted curve (red dotted line) (A); the weight loss curves of TGG and TGG/LN/NGF (B). The values are represented as mean ± SD (*n* = 4).

NGF was released from the gellan gum mainly through the following steps. First, the NGF located in the hydrogel shallow layer and on the surface permeated into the medium. Subsequently, the NGF inside the hydrogel diffused to the surface of the hydrogel under the action of the concentration gradient. Finally, NGF desorbed on the surface of the hydrogel and diffused into the medium. The drug release process was accompanied by complex physical and chemical reactions. Thus, the drug release model could be better understood by studying the kinetics of drug release. From the release curve, it was inferred that NGF was released relatively fast in the first 20 h and the cumulative release reached 63.02%. From Table 1, we can infer that the *R*^2^ values for the zero-order, first-order, Higuchi, and Korsmeyer–Peppas models are 0.93879, 0.94235, 0.98964, and 0.9901, respectively. The Korsmeyer–Peppas model is used to simulate the drug release kinetics of biodegradable systems. The parameter *n* is the release exponent that can explain the release mechanism of a drug delivery system. When *n* < 0.5, the drug release mechanism belongs to a Fickian diffusion-controlled release mechanism. Because of the dynamic disulfide bond crosslinking structure of the TGG hydrogel, the Korsmeyer–Peppas diffusion index (*n* = 0.484) was less than 0.5 and the release model belonged to Fickian diffusion-controlled.^28^ This NGF release behavior suggested that NGF could be slowly released from the TGG hydrogel into an ambient environment while retaining a portion in the hydrogel interior for neuronal stem cell growth. Fig. 2B shows the degradation properties of TGG and TGG/LN/NGF. It can be seen from the weight loss curve that the weights of both TGG and TGG/LN/NGF hydrogels decrease slowly within 10 days, guaranteeing stabilized NGF release in the early phase. Ten days later, the weight decreased relatively faster. The overall degradation rate of TGG and TGG/LN/NGF displayed no significant difference. The sustained and stable degradation behavior provides a safe guarantee for NGF release and implantation *in vivo*.

**Table 1:** The release kinetic models of the NGF-based TGG hydrogels

Drug	Dose	Medium	*R* ^2^				*n*
			Zero-order	First-order	Higuchi	Korsmeyer–Peppas	
NGF	200 ng mL^−1^	PBS	0.93879	0.94235	0.98964	0.9901	0.484

### 3.3 Proliferation and synergy effect of neuronal stem cells

**Fig. 3:**
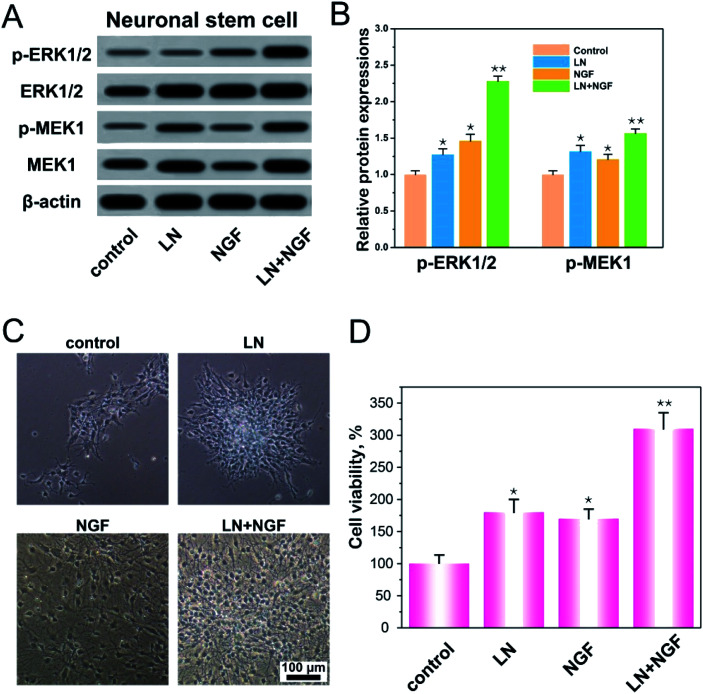
Western blot analysis of the protein expression of ERK/MAPK pathway-related proteins in neuronal stem cells (A); the relative amounts of p-ERK1/2 and p-MEK1 (B); the proliferation of neuronal stem cells (C); the cell viability assayed by CCK-8 (D). All the experiments were conducted with the GG control group, LN group, NGF group, and LN + NGF group at 72 h. *p < 0.05, **p < 0.01 compared to the GG control group. The scale bar is 100 μm. The values are represented as mean ± SD (*n* = 4).

LN, as an important component of the extracellular matrix, plays a regulatory role in nerve regeneration.^29^ The first step in nerve regeneration is supporting the survival environment for the proliferation of neuronal stem cells. LN can interact with integrins on the cell membrane to promote focal adhesion, and the downstream protein focal adhesion kinase (FAK) of focal adhesion can activate the ERK–MAPK pathway.^30,31^ NGF has also been shown to activate the MAPK signaling pathway.^32^ As a consequence, we speculated that LN and EGF could synergistically promote the ERK–MAPK signaling pathway to promote the proliferation of neural stem cells. We verified the expressions of p-ERK1/2 and p-MEK1 on the classical MAPK signaling pathway (Fig. 3A and B). From the data in the figures, both LN and NGF can promote the phosphorylation of p-ERK1/2 and p-MEK1 independently. With LN treatment only, the relative amounts of p-ERK1/2 and p-MEK1 were 1.27- and 1.32-fold higher than the control group. With NGF treatment only, the relative amounts of p-ERK1/2 and p-MEK1 were 1.46- and 1.20-fold higher than the control group. When LN was combined with NGF simultaneously, the relative amounts of p-ERK1/2 and p-MEK1 were 2.28- and 1.56-fold higher than the control group, and the ERK–MAPK signal was significantly enhanced (**p < 0.01). Fig. 3C shows the proliferation of neuronal stem cells. After culturing, in the control group, most of the neuronal stem cells were relatively scattered. The cells treated with LN grew into a ball of nerves. The cells in the NGF treatment group were evenly dispersed, but the proliferation was significantly smaller than that in the LN and NGF treatment groups. The cells treated by LN and NGF migrated from the nerve bulb to the surrounding area and produced tiny axons and had the largest number. Fig. 3D shows the cell proliferation data at 72 h *via* the CCK-8 method; the quantities of neuronal stem cells treated with LN, NGF, and LN + NGF were 182.3 ± 17.9-, 169.2 ± 16.1-, and 313.7 ± 24.2-fold higher than the control group (100 ± 14.6), indicating that LN combined with NGF could significantly enhance the proliferation ability of neuronal stem cells (**p < 0.01). This could promote the ERK–MAPK signal pathway.

### 3.4 Apoptosis process of neuronal stem cells induced by the TGG gels

**Fig. 4:**
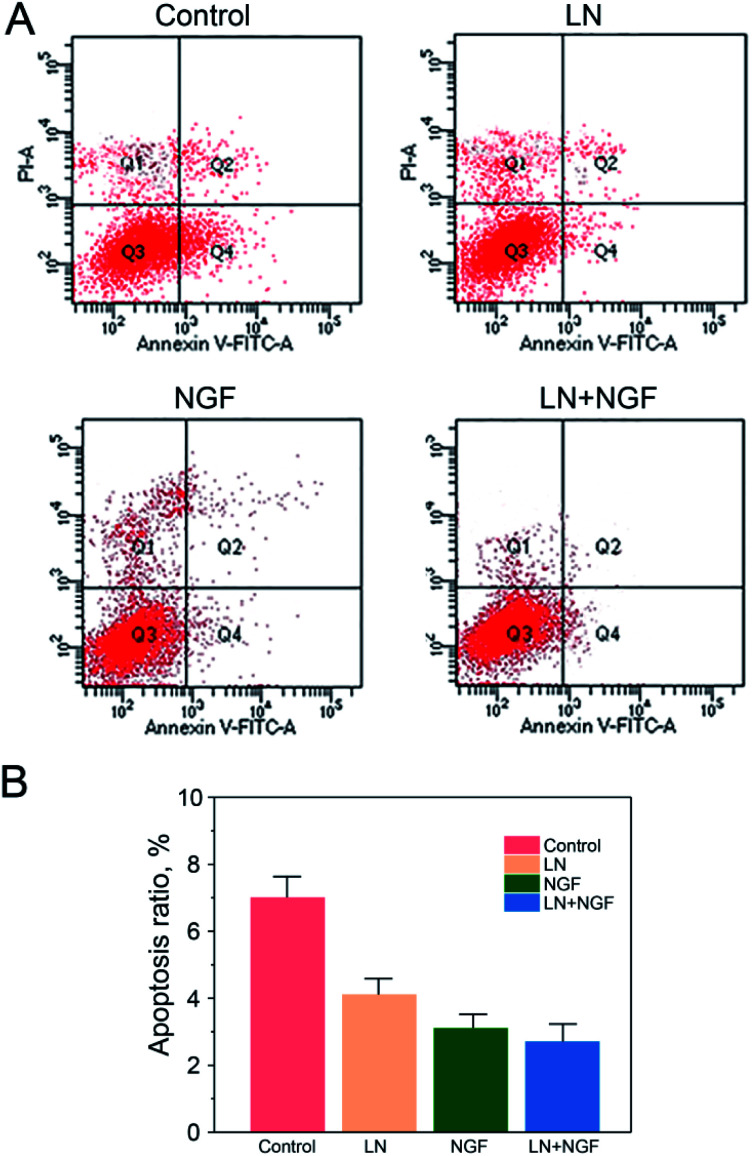
Flow cytometry apoptosis data (A) and statistical data (B) of neuronal stem cells after treatment with the GG control group, LN group, NGF group, and LN + NGF group for 24 h (A). The values are represented as mean ± SD (*n* = 3).

The effect of the four TGG gels on apoptosis was investigated by Annexin V and PI double staining and analyzed *via* flow cytometry (Fig. 4). Neuronal stem cells cultured with the TGG gel displayed no obvious apoptosis phenomenon with a total apoptosis ratio of 8.41% (a sum of the early apoptosis ratio of 6.25% and the late apoptosis ratio of 2.16%). When the cells were treated with both LN and NGF, there was a 2.69% apoptosis ratio, which was lower than that for the LN- (4.08%) or NGF (3.14%)-treated group. This again confirmed the enhanced proliferation efficacy *in vitro via* the surface functionalization of LN and loading NGF.

This can be due to the biological action of LN. LN has the three-peptide sequence of “Arg–Gly–Asp”, which can be combined tightly with the integrin on the cytomembrane. The neuronal stem cells have to be attached to the extracellular matrix, such as LN, to grow and proliferate. If they cannot be attached to the extracellular matrix, they will stop growing and proliferating, finally resulting in death. Therefore, it is significant to employ LN to modify the TGG gel to enhance the attachment and proliferation of neuronal stem cells.

### 3.5 Differentiation of neuronal stem cells

**Fig. 5:**
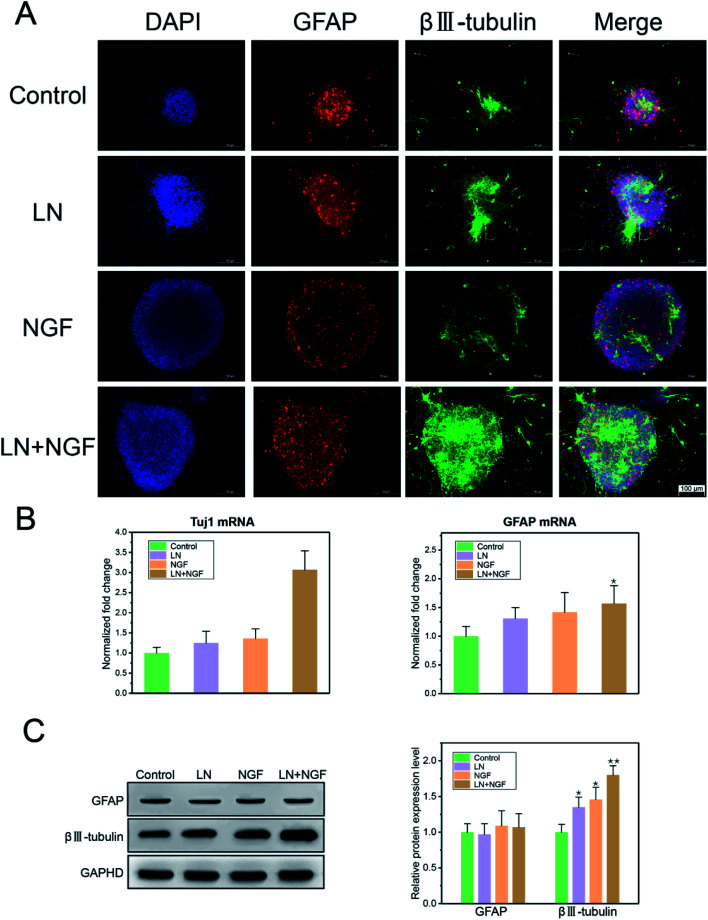
The CLSM image of neuronal stem cell differentiation in TGG hydrogels. Neurons were stained by βIII–tubulin, glial cells were stained by GFAP, and the nucleus was stained with DAPI. The scale bar is 100 μm (A). The q-PCR relative quantitative analysis results of GFAP and Tuj1 (B) and western blot images and relative quantitative analysis results of GFAP and βIII–tubulin (C). All the experiments were conducted with the GG control group, LN group, NGF group, and LN + NGF group for 1 week. The values are represented as mean ± SD (*n* = 4).

As GFAP and βIII–tubulin are the biomarkers of glial cells and neurons, respectively, neuronal stem cell differentiation behavior was determined using the immunofluorescence staining of GFAP and βIII–tubulin. Neural stem cells were cultured in TGG, LN, NGF, and LN + NGF gels for one week. Fig. 5A shows the immunofluorescence results in the three-dimensional culture environment of neuronal stem cells. The differentiated neural stem cells showed both βIII–tubulin, GFAP expression positive. The merge channel showed that glial cells and neurons mixed and grew together in the hydrogel interior, indicating the excellent biocompatibility of the hydrogel for glial cells and neurons. The merge channel also showed that the neural stem cells in the four-dimensional culture systems had neural differentiation ability, due to which they could differentiate neurons and glial cells. At the same time, the neural stem cells cultured by LN combined with NGF showed better differentiating ability than the stem cells cultured by LN or NGF alone. In the LN + NGF group, more green fluorescence was observed, indicating that LN and NGF facilitated neural stem cells to differentiate into more neurons. This suggests that LN and NGF can still play a synergistic role in promoting stem cell proliferation and differentiation in a three-dimensional environment. To further quantitatively analyze the proportion of glial cells and nerve cells relatively accurately, q-PCR and western blot analyses were applied to analyze the marker genes and proteins of glial cells and neurons. Fig. 5A shows the q-PCR relative quantitative analysis results of GFAP and Tuj1 after one week. The mRNA level of Tuj1 was significantly improved for the LN + NGF group compared to that for the control, LN, and NGF groups (**p < 0.01). However, the mRNA level of GFAP showed limited enhancement. Fig. 5B shows the western blot images and relative quantitative analysis results of GFAP and βIII–tubulin after one week. The expression of Tuj1 was significantly enhanced for the LN + NGF group compared to that for the control, LN, and NGF groups (**p < 0.01). However, the expression of GFAP showed no significant change. Immunofluorescence staining combined with q-PCR and western blot analyses demonstrated that LN combined with NGF could induce neuronal stem cells to differentiate into neuronal cells. This remarkable enhancement effect for neuron cell growth onto TGG was due to the adhesion effect of LN and the stimulating effect of TGG. In addition, the NGF-loaded hydrogel exhibited a slow release effect and could maintain its biological activity in facilitating neuron growth for a long time.

## 4 Ethical statement

All animal procedures were performed in accordance with the Guidelines for the Care and Use of Laboratory Animals of “Jinan University” and approved by the Animal Ethics Committee of “Jinan University.

## 5 Conclusion

An NGF slow release system was prepared by modifying TGG. TGG was characterized by ^1^H NMR spectroscopy and scanning electron microscopy, and its rheological behavior was studied. The NGF release curve fitted the Korsmeyer–Peppas model and belonged to a Fickian diffusion-controlled release mechanism. Furthermore, the neuronal stem cells interweaved and formed a network completely with oval or triangular morphology in TGG. In this study, a three-dimensional scaffold prepared by gellan gum had a structure similar to the internal microenvironment of nerve cell growth, thus promoting the orientation differentiation of neurons. The material reported here is a potential biological material for neuronal stem cell tissue engineering and may provide a new approach for exploring the effective treatment of neuronal deficiency and nervous system trauma.

## Conflicts of interest

There are no conflicts to declare.

## Supplementary Material
